# Improving access to dementia care community resources in the Pacific island of Guam

**DOI:** 10.1002/alz.086370

**Published:** 2025-01-09

**Authors:** Iain K. B. Twaddle, Nikolas Jude Gutierrez, Maree Josephine Saloma, Camille Minerva Maestrecampo

**Affiliations:** ^1^ University of Guam, Mangilao Guam

## Abstract

The island of Guam is a U.S. territory in the Western Pacific with a population of approximately 174,000. Most persons with Alzheimer’s disease and related dementias (ADRD) in Guam live at home with their family. This is due, in part, to Guam’s indigenous Chamorro culture, which emphasizes the importance of caring for the *manåmko’* (elderly), but also to the limited availability of residential care facilities for older adults. In fact, Guam currently has no nursing homes or assisted living facilities that specialize in care for persons living with ADRD. This places a heavy burden on Guam’s families. Fortunately, Guam has an impressive array of community‐based resources to support persons living with dementia and their family caregivers. However, many members of the community are not aware of these services or do not know how to access them. To improve access to Guam’s dementia care support services, a series of education outreach presentations were provided to the community through an online support group for family caregivers of persons with dementia held weekly on Wednesday evenings and Saturday mornings. The presentations were conducted by elder care specialists from local government agencies and private healthcare organizations. A wide range of services were covered including: (1) adult day care services; (2) transportation services; (3) congregate and home‐delivered meals; (4) adult protective services; (5) case management services; (6) in‐home services; (7) home health services; (8) long‐term residential care for non‐ambulatory older adults; (9) government health insurance programs for older adults including Medicaid and Guam’s Medically Indigent Program; and (10) legal services for older adults, including guardianship, powers of attorney, wills, living wills, and advance healthcare directives. Each presentation provided guidance on eligibility criteria and application procedures, as well as contact information for key personnel. Program outcomes indicate the following: (1) all sessions were well attended; (2) participant feedback was overwhelmingly positive; and (3) a significant number of participants followed through with applying for dementia care services. In sum, education outreach presentations conducted online can serve as an effective means to improve access to community resources for persons living with ADRD and their family caregivers.

